# Secure and Efficient Three-Factor Protocol for Wireless Sensor Networks

**DOI:** 10.3390/s18124481

**Published:** 2018-12-18

**Authors:** Jihyeon Ryu, Hakjun Lee, Hyoungshick Kim, Dongho Won

**Affiliations:** 1 Department of Platform Software, Sungkyunkwan University, Gyeonggi-do 16419, Korea; jhryu@security.re.kr; 2 Department of Electrical and Computer Engineering, Sungkyunkwan University, Gyeonggi-do 16419, Korea; hjlee@security.re.kr; 3 Department of Computer Engineering, Sungkyunkwan University, Gyeonggi-do 16419, Korea; hyoung@skku.edu

**Keywords:** wireless sensor networks, user authentication, biometric, smart card

## Abstract

Wireless sensor networks are widely used in many applications such as environmental monitoring, health care, smart grid and surveillance. Many security protocols have been proposed and intensively studied due to the inherent nature of wireless networks. In particular, Wu et al. proposed a promising authentication scheme which is sufficiently robust against various attacks. However, according to our analysis, Wu et al.’s scheme has two serious security weaknesses against malicious outsiders. First, their scheme can lead to user impersonation attacks. Second, user anonymity is not preserved in their scheme. In this paper, we present these vulnerabilities of Wu et al.’s scheme in detail. We also propose a new scheme to complement their weaknesses. We improve and speed up the vulnerability of the Wu et al. scheme. Security analysis is analyzed by Proverif and informal analysis is performed for various attacks.

## 1. Introduction

A wireless sensor network (WSN) is a distributed network of autonomous sensors that are typically used to collect information about environmental or physical conditions. Wireless sensor networks are applicable to a variety of applications such as environmental monitoring, health care, smart grid and surveillance [1,2,3,4,5,6] because they can be easily deployed without a significant cost penalty.

In general, a WSN system consists of four entities: (1) user interface, (2) a sensor node that measures physical or environmental conditions, (3) a gateway node that forwards the information received from the sensor nodes to a central server, and (4) a central server that collects the information from the sensor nodes and analyze it. Naturally, however, the security of WSN is critical because network packets can be easily captured and modified in WSN due to the inherent characteristics of wireless networks. Therefore, we need to provide security protocols in order to ensure security properties such as confidentiality, integrity, and authenticity even when data packets on a WSN are captured and modified in an unauthorized manner.

Due to the inherent weakness of WSNs, many researchers have proposed security protocols to achieve fundamental security goals of WSNs. As one of the pioneers in this area, Watro et al. [7] proposed a security protocol using RSA (See Table A1 for details) for wireless sensor networks. To enhance the security of the authentication procedure, Das [2] extended their protocol to a two-factor user authentication protocol for WSNs where a user has to hold both a password and smartcard. Because their proposed authentication scheme provides reasonable security properties, it has been widely used for WSNs as a de-factor standard protocol [8,9,10]. However, He et al. [11] found that Das’s protocol is vulnerable to several attacks such as insider attacks, impersonation attacks and lack of secure mutual authentication. They also suggested an authentication scheme by fixing the discovered problems. However, Kumar et al. [12] also discovered several security flaws such as information leakage, no session key agreement, no mutual authentication, and lack of anonymity in Das’s protocol.

Recently, some researchers (e.g., [13]) have started to develop user authentication schemes for WSNs using ECC, which can provide the same security as RSA with a smaller key size. ECC is the most efficient algorithm that satisfies forward secrecy and backward secrecy among the algorithms so far. Xue et al. [14] particularly introduced a temporal-credential-based protocol to provide user anonymity. However, Jiang et al. [15] demonstrated that Xue et al.’s scheme has four critical security flaws: (1) identity guessing attacks, (2) online password guessing attacks by privileged insiders, and (3) offline password guessing attacks with a victim’s smartcard. Jiang et al. also suggested a new authentication scheme to address their discovered issues.

More recently, Das [16] found that Jiang et al. [15]’s scheme has significant security issues such as the vulnerabilities to insider and de-synchronization attacks and lack of formal security proof of the proposed scheme. To address these issues, Das proposed several three-factor user authentication schemes [16,17,18] by introducing a new factor of user biometrics. Again, Wu et al. [1] found that all the Das’ schemes [16,17,18] are vulnerable to de-synchronization and offline password guessing attacks. In addition, the protocols [17,18] are vulnerable to user impersonation and offline password guessing attacks. To fix such problems, Wu et al. [1] suggested a three-factor user authentication scheme using ECC for WSNs.

In this paper, however, we found that Wu et al.’s scheme [1] has two security flaws against outsider attackers. First, their scheme can lead to user impersonation attacks. Second, user anonymity is not preserved because the user identity can be revealed from an anonymous login request message. We will explain these in the reminder of this paper. Our key contributions are summarized below:

- We discovered two security weaknesses in Wu et al.’s scheme [1], which was recently designed for user authentication using ECC in WSN systems. We demonstrated that a malicious outsider holding a smart card can extract the secret parameters from his/her smart card; the extracted secret parameters can be used to perform impersonation attacks and reveal the identity of the user from a login request message.
- We also proposed a novel three-factor user authentication scheme for WSN by extending Wu et al.’s scheme [1]. The proposed authentication scheme not only accomplishes several important security properties but also improves the performance of the protocol in time.

The rest of the paper is structured as follows: Section 2 gives some preliminaries of the cryptographic primitives (i.e., ECC and fuzzy extractor) used in our paper and explains the threat model and assumptions. Section 3 provides a review of Wu et al.’s scheme [1]. Section 4 analyzes the security weaknesses of their scheme. Section 5 presents a novel three-factor user authentication scheme by fixing security issues in Wu et al.’s scheme. Section 6 and Section 7 provide security and performance analysis results, respectively. We conclude in Section 8.

## 2. Preliminaries

In this section, we introduce elliptic curves, fuzzy extractors, and threat models to be used in this paper.

### 2.1. Elliptic Curve Cryptosystem

The Elliptic curve cryptosystem (ECC) is the most frequently used password system in modern passwords and has strong security characteristics. Miller [19] and Neal [20] create ECC in 1985 and 1987, respectively. ECC uses the following formula:

(1) $$y^{2}=x^{3}+ax+b\;mod\;p\;a,b\in F_{p}.$$

The above equation is ECC on the $F_{p}$. The following conditions must be met in order to ensure safety:

(2) $$4a^{3}+27b^{2}\neq 0\;mod\;p.$$

This is a formula that guarantees the non-singularity of an elliptic curve. When using this elliptic curve, safety is ensured as follows:

1. Elliptic Curve Computational Diffie–Hellman Problem (ECCDHP): Given xyP, it is impossible to find xP, yP.
2. Elliptic Curve Decisional Diffie–Hellman Problem (ECDDHP): Given xP, yP it is impossible to find xyP.
3. Elliptic Curve Discrete Logarithm Problem (ECDLP): Given *P*, xP it is impossible to find *x*.

We hypothesized that *P* is the point on $F_{p}$, xP is the result of calculating *P* times *x*, yP is the result of calculating *P* times *y*, and xyP is the result of calculating *P* times xy.

### 2.2. Fuzzy Extractor

The user’s biometric information is very important information. In general, human biometric recognition is perceived differently each time, and the fuzzy extractor plays a role in correcting it. The fuzzy extractor can obtain a unique string using error tolerance. The fuzzy extractor is operated through two procedures (Gen, Rep), demonstrated as [17,21]:

(3) $$Gen\;\left(B\right)\to \langle \alpha ,\beta \rangle ,$$

(4) $$Rep\;(B^{*},\beta )=\alpha .$$

Gen is a probabilistic generation function for which the biometrics *B* returns a factored out string $\alpha \in {\left\{0,1\right\}}^{k}$ and a coadjutant string $\beta \in {\left\{0,1\right\}}^{*}$, and Rep is a function that restores β to α, and any vector $B^{*}$ close to *B* [22].

### 2.3. Threat Assumption

We introduce a threat model [8], and consider constructing the threat assumptions as follows:

1. The attacker A can be a user, a gateway, or a sensor. Any registered user can act as an attacker.
2. A can intercept or eavesdrop on all communication messages in a public channel, thereby capturing any message exchanged between a user and gateway or sensor.
3. A has the ability to modify, reroute, or delete the intercepted message.
4. Stored parameters can be extracted from smart cards using the side channel attack [23].
5. An external attacker A (outsider) can also register, login and receive his/her smart card.

## 3. Review of Wu et al.’s Scheme

In this section, we perform an analysis on Wu et al.’s scheme in order to scrutinize the security weakness of their scheme in the next section. Wu et al.’s scheme consists of four phases: registration phase, login phase, authentication phase, and password change phase. In addition, it applies ECC such as the [17] schemes. To begin with, GWN creates *G* on *E* $(F_{p})$ with *P* as a generator and large prime *n* as an order. After that GWN picks a private key *x* under two hash functions *h* (·), $h_{1}$ (·) and security length $l_{s}$. In their scheme, they assume that the length of all random numbers should be above $l_{s}$. Other notations used in Wu et al.’s scheme are abridged in Table 1.

### 3.1. Registration Phase

Registration phase is divided into two parts: user registration phase and registration phase.

#### 3.1.1. User Registration

1. The user $U_{i}$ first decides his/her identification $ID_{i}$ and password $PW_{i}$. With a random number $r_{i}$, it imprints $B_{i}$ over a device for biometrics collection, and calculates $Gen(B_{i})=(R_{i}$, $P_{bi})$, $DID_{i}=h(ID_{i}∥r_{i})$ and $HPW_{i}=h(PW_{i}∥r_{i}∥R_{i})$. He/she then requests the registration message $\{ID_{i}$, $DID_{i}\}$ to the gateway node GWN over a secure channel.
2. After the registration request message from the $U_{i}$ is received, GWN computes $B_{1}^{\prime }=h(DID_{i}∥x),$ where *x* is GWN’s secret key, prepares a smart card for $U_{i}$ containing *h* (·), $h_{1}$ (·), *P*, and collects $ID_{i}$ in the database. The next thing is that GWN sends the smart card with $B_{1}^{\prime }$ to the $U_{i}$ securely.
3. When receiving the smart card with $B_{1}^{\prime }$ from the GWN, $U_{i}$ computes $B_{1}=B_{1}^{\prime }\;⊕\;HPW_{i}$ and $B_{2}=h(ID_{i}∥R_{i}∥PW_{i})\;⊕\;r_{i}$ with storing $B_{1}$, $B_{2}$, *P* and $P_{bi}$ in the smart card.

#### 3.1.2. Sensor Registration

1. GWN determines an identity SIDj for new sensor node $S_{j}$, computes hash function $c_{j}=h$ $(SID_{j}∥x)$, and sends $\left\{SID_{j},c_{j}\right\}$ to $S_{j}$.
2. $S_{j}$ stores *P*, $SID_{j}$ and $c_{j}$, and enters the WSN.

### 3.2. Login Phase

1. $U_{i}$ enters $ID_{i}$, $PW_{i}$ and $B_{i}^{\prime }$. Then, the smart card computes $Rep(B_{i}^{\prime }$, $P_{bi})=R_{i}$, $r_{i}=B_{2}\;⊕\;h$ $(ID_{i}∥R_{i}∥PW_{i})$, $HPW_{i}=h$ $(PW_{i}∥r_{i}∥R_{i})$ and $DID_{i}=h$ $(ID_{i}∥r_{i})$.
2. The smart card produces random numbers $r_{i}^{new}$, $e_{i}$ and α∈[1, n−1], and selects a special sensor $SID_{j}$. Then, the smart card calculates $DID_{i}^{new}=h$ $(ID_{i}∥r_{i}^{new})$, $C_{1}=B_{1}\;⊕\;HPW_{i}\;⊕\;e_{i}$, $C_{2}=\alpha P$, $C_{3}=h$ $(e_{i})\;⊕\;DID_{i}^{new}$, $Z_{i}=ID_{i}\;⊕\;h$ $(e_{i}∥DID_{i})$ and $C_{4}=h$ $(ID_{i}∥e_{i}∥DID_{i}∥DID_{i}^{new}∥C_{2}∥SID_{j})$. The value $C_{4}$ is used to certify the integrity of the identities and the new data generated by the user side as well as to authenticate the source of the message $M_{1}$.
3. $U_{i}$ sends the login request messages $M_{1}=\{C_{1}$, $C_{2}$, $C_{3}$, $C_{4}$, $Z_{i}$, $DID_{i}$, $SID_{j}\}$ to GWN.

### 3.3. Authentication Phase

1. After the login request messages $M_{1}$ arrives from the user $U_{i}$, GWN first computes $e_{i}=C_{1}\;⊕\;h$ $(DID_{i}∥x)$, $DID_{i}^{new}=C_{3}\;⊕\;h$ $(e_{i})$ and $ID_{i}=Z_{i}⊕h$ $(e_{i}∥DID_{i})$, and verifies the legitimacy of $ID_{i}$ and $C_{4}\overset{?}{=}h$ $(ID_{i}∥e_{i}∥DID_{i}∥DID_{i}^{new}∥C_{2}∥SID_{j})$. GWN terminates the session if either verification fails. If three failures continuously occur in a certain time span as defined, $U_{i}$’s account will be frozen; otherwise, GWN calculates $c_{j}=h$ $(SID_{j}∥x)$ and $C_{5}=h$ $(c_{j}∥DID_{j}∥SID_{j}∥C_{2})$ and sends $M_{2}=\{C_{2}$, $C_{5}$, $DID_{i}\}$ to the sensor node $S_{j}$. The value $C_{5}$ is used to accredit the integrity of the strings containing $c_{j}$, and the data can be used for the sensor $S_{j}$ to acquire the correct data for calculating the session key. This is also done for verification of the source of $M_{2}$.
2. $S_{j}$ checks the validity of $C_{5}$, $C_{5}\overset{?}{=}h$ $(c_{j}∥DID_{i}∥SID_{j}∥C_{2})$ with its identity $SID_{j}$. If this step fails, $S_{j}$ will terminate the session. Otherwise, $S_{j}$ then chooses β∈[1, n−1] and calculates $C_{6}=\beta P$, $sk_{s}=\beta C_{2}$, $C_{7}=h_{1}$ $(C_{2}∥C_{6}∥sk_{s}∥DID_{i}∥SID_{j})$ and $C_{8}=h$ $(DID_{i}∥SID_{j}∥c_{j})$. The main functionality of $C_{7}$ is used for checking the integrity of the session key and $C_{6}$, which is needed by $U_{i}$ to compute the session key. Both $C_{7}$ and $C_{8}$ are also used to validate the source of $M_{3}$. In the end, $S_{j}$ sends $M_{3}=\{C_{6}$, $C_{7}$, $C_{8}\}$ to GWN.
3. GWN checks $C_{8}\overset{?}{=}h$ $(DID_{i}∥SID_{j}∥c_{j})$. If the validation phase fails, GWN terminates the session; otherwise, GWN computes $C_{9}=h$ $(DID_{i}^{new}∥x)⊕h$ $(DID_{i}∥e_{i})$ and $C_{10}=h$ $(ID_{i}∥SID_{j}∥DID_{i}∥DID_{i}^{new}∥e_{i}∥C_{9})$. The value $C_{10}$ is to check the validation of the source’s message $M_{4}$. Eventually, GWN sends the message $M_{4}=\{C_{6}$, $C_{7}$, $C_{9}$, $C_{10}\}$ to $U_{i}$.
4. $U_{i}$ checks $C_{10}\overset{?}{=}h$ $(ID_{i}∥SID_{j}∥DID_{i}∥DID_{i}^{new}∥e_{i}∥C_{9})$. $U_{i}$ then computes the session key $sk_{u}=\alpha C_{6}$, and checks $C_{7}\overset{?}{=}h_{1}$ $(C_{2}∥C_{6}∥sk_{u}∥DID_{i}∥SID_{j})$. $U_{i}$ terminates the session if $U_{i}$ fails the verification phase. Otherwise, $U_{i}$ computes $HPW_{i}^{new}=h$ $(PW_{i}∥r_{i}^{new}∥R_{i})$, $B_{1}^{new}=C_{9}\;⊕\;h$ $(DID_{i}∥e_{i})\;⊕\;HPW_{i}^{new}$ and $B_{2}^{new}=h$ $(ID_{i}∥R_{i}∥PW_{i})\;⊕\;r_{i}^{new}$, and replaces $(B_{1}$, $B_{2})$ with $(B_{1}^{new}$, $B_{2}^{new})$ in each smart card separately.

### 3.4. Password and Biometrics Change Phase

1. Same as the step 1 in the Login phase.
2. The smart card produces random numbers $r_{i}^{new}$ and $e_{i}$, calculates $DID_{i}^{new}$, $C_{1}$, $C_{3}$, $Z_{i}$ and $C_{11}=h$ $(ID_{i}∥e_{i}∥DID_{i}∥DID_{i}^{new})$, and sends $M_{5}=\{C_{1}$, $C_{3}$, $Z_{i}$, $C_{11}$, $DID_{i}\}$ with a password change request to GWN. The value $C_{11}$ is similar to $C_{4}$, which is to confirm the integrity of the identities as well as to verify the source of $M_{5}$.
3. GWN obtains $e_{i}$, $ID_{i}$ and $DID_{i}^{new}$ as in step 1 of the authentication phase, and checks $ID_{i}$ and $C_{11}\overset{?}{=}h$ ($ID_{i}∥e_{i}∥DID_{i}∥DID_{i}^{new})$. If the verification stage fails, GWN terminates the session; otherwise, GWN computes $C_{9}=h$ ($DID_{i}^{new}∥x)\;⊕\;h$ ($DID_{i}∥e_{i})$ and $C_{12}=h$ ($ID_{i}∥DID_{i}∥DID_{i}^{new}∥e_{i}∥C_{9})$ and sends $M_{6}=\{C_{9}$, $C_{12}\}$ and a grant to $U_{i}$. Here, $C_{12}$ is to verify the source of $M_{6}$.
4. $U_{i}$ checks $C_{12}\overset{?}{=}h$ ($ID_{i}∥DID_{i}∥DID_{i}^{new}∥e_{i}∥C_{9})$. If two values are not equal, then $U_{i}$ terminates this session; otherwise, $U_{i}$ inputs a new password $PW_{i}^{new}$ and a new biometric information $B_{i}^{new}$. The next thing is that the smart card computes Gen ($B_{i}^{new})=(R_{i}^{new}$, $P_{bi}^{new})$, $HPW_{i}^{new2}=h$ ($PW_{i}^{new}∥r_{i}^{new}∥R_{i}^{new})$, $B1^{new2}=C_{9}\;⊕\;h$ ($DID_{i}∥e_{i})\;⊕\;HPW_{i}^{new2}$ and $B_{2}^{new2}=h$ ($ID_{i}∥R_{i}^{new}∥PW_{i}^{new})\;⊕\;r_{i}^{new}$. Finally, $U_{i}$ substitutes $(B_{1}^{new2}$, $B_{2}^{new2}$, $P_{bi}^{new2})$ for $(B_{1}$, $B_{2}$, $P_{bi})$ in the smart card, respectively.

## 4. Cryptanalysis of Wu et al.’s Scheme

We show that Wu et al.’s scheme [1] possesses certain some security vulnerabilities in this section. The following problems have been found and are described in detail below.

### 4.1. Extract Critical Information

1. An attacker A who is a legitimate user and he/she can own his/her smart card. The smart card can extract the value $\{B_{1A}$, $B_{2A}$, *P*, $P_{bA}\}$.
2. A can thus obtain *h* ($DID_{A}∥x)=B_{1A}\;⊕\;HPW_{A}$, and use this variable for other attacks because this value is a critical value that be used on the user identification in the GWN.

### 4.2. No User Anonymity

Attacker A can extract the identity of $U_{i}$ from the login request message $M_{i}$ of $U_{i}$. Assume that A eavesdrops on the login request message $M_{1}=\{C_{1}$, $C_{2}$, $C_{3}$, $C_{4}$, $Z_{i}$, $DID_{i}$, $SID_{j}\}$ of $U_{i}$. We also assume that attacker A has *h* ($DID_{A}∥x)$ through 5.1. Extract Critical Information. The details are as follows:

1. Attacker A first generates random numbers $r_{A}^{new}$, $e_{A}$, and $\alpha_{A}\in [1$, n−1], and selects a special sensor $SID_{j}$. $C_{1A}=B_{1A}\;⊕\;HPW_{A}\;⊕\;e_{A}$, $C_{2A}=\alpha_{A}P$, $C_{3A}=h$ ($e_{A})\;⊕\;DID_{i}$, $Z_{A}=ID_{A}\;⊕\;h$ ($e_{A}∥DID_{A})$ and $C_{4A}=h$ ($ID_{A}∥e_{A}∥DID_{A}∥DID_{i}∥C_{2A}∥SID_{j})$.
2. *A* forwards the login request message $M_{1A}=\{C_{1A}$, $C_{2A}$, $C_{3A}$, $C_{4A}$, $Z_{A}$, $DID_{A}$, $SID_{j}\}$ to the gateway node GWN.
3. After receiving the login request message from A, GWN computes $e_{A}=C_{1A}\;⊕\;h$ ($DID_{A}∥x)$, $DID_{i}=C_{3A}\;⊕\;h$ ($e_{A})$ and $ID_{A}=Z_{A}\;⊕\;h$ ($e_{A}∥DID_{A})$, and checks the validity of $ID_{A}$ and $C_{4A}\overset{?}{=}h$ ($ID_{A}∥e_{A}∥DID_{A}∥DID_{i}∥C_{2A}∥SID_{j})$. GWN then computes $c_{j}=h$ ($SID_{j}∥x)$ and $C_{5A}=h$ ($c_{j}∥DID_{j}∥SID_{j}∥C_{2A})$ and sends $M_{2A}=\{C_{2A}$, $C_{5A}$, $DID_{A}\}$ to $S_{j}$.
4. $S_{j}$ checks $C_{5A}\overset{?}{=}h$ ($c_{j}∥DID_{A}∥SID_{j}∥C_{2A})$ with its identity $SID_{j}$. If this does not hold, $S_{j}$ terminates the session. $S_{j}$ then selects $\beta_{A}\in [1$, n−1] and computes $C_{6A}=\beta_{A}P$, $sk_{s}=\beta_{A}C_{2A}$, $C_{7A}=h_{1}$ ($C_{2A}∥C_{6A}∥sk_{s}∥DID_{A}∥SID_{j})$ and $C_{8A}=h$ ($DID_{A}∥SID_{j}∥c_{j})$. $S_{j}$ sends $M_{3A}=\{C_{6A}$, $C_{7A}$, $C_{8A}\}$ to GWN.
5. GWN tests $C_{8A}\overset{?}{=}h$ ($DID_{A}∥SID_{j}∥c_{j})$. If this does not hold, GWN terminates the session; otherwise, GWN calculates $C_{9A}=h$ ($DID_{i}∥x)\;⊕\;h$ ($DID_{A}∥e_{A})$ and $C_{10A}=h$ ($ID_{A}∥SID_{j}∥DID_{A}∥DID_{i}∥e_{A}∥C_{9A})$. Finally, GWN sends the message $M_{4A}=\{C_{6A}$, $C_{7A}$, $C_{9A}$, $C_{10A}\}$ to attacker A.
6. A calculates *h* ($DID_{i}∥x)=h$ ($DID_{A}∥e_{A})\;⊕\;C_{9A}$. Now, A can compute $e_{i}=C_{1}\;⊕\;h$ ($DID_{i}∥x)$. Eventually, A can find $ID_{i}=h$ ($e_{i}∥DID_{i})\;⊕\;Z_{i}$.

This result shows that Wu et al.’s scheme does not ensure user anonymity.

### 4.3. User Impersonation Attack

An attacker A can impersonate any user through the identity of others and his/her own information. We assume the casualty is $U_{i}$. We also assume that attacker A has *h* ($DID_{A}∥x)$ through Section 5.1. Extract Critical Information. The detailed method is as follows:

1. Attacker A selects $ID_{i}$ who is the target of the user impersonation attack.
2. A selects random numbers $r_{A}^{new}$, $e_{A}$, and $\alpha_{A}\in [1$, n−1] and selects a particular sensor $SID_{j}$. Then, A calculates $DID_{A}^{new}=h$ ($ID_{A}∥r_{A}^{new})$, $C_{1A}=B_{1A}\;⊕\;HPW_{A}\;⊕\;e_{A}$, $C_{2A}=\alpha_{A}P$, $C_{3A}=h$ ($e_{A})\;⊕\;DID_{A}^{new}$, $Z_{A}=ID_{i}\;⊕\;h$ ($e_{A}∥DID_{A})$ and $C_{4A}=h$ ($ID_{i}∥e_{A}∥DID_{A}∥DID_{A}^{new}∥C_{2A}∥SID_{j})$. $C_{4A}$ is to check the new data produced on the user side and the integrity of the identities as well as to verify the source of $M_{1A}$.
3. *A* forwards the login request message $M_{1A}=\{C_{1A}$, $C_{2A}$, $C_{3A}$, $C_{4A}$, $Z_{A}$, $DID_{A}$, $SID_{j}\}$ to GWN.
4. After obtaining the message from the A, GWN calculates $e_{A}=C_{1A}\;⊕\;h$ ($DID_{A}∥x)$, $DID_{A}^{new}=C_{3A}\;⊕\;h$ ($e_{A})$ and $ID_{i}=Z_{A}\;⊕\;h$ ($e_{A}∥DID_{A})$, and checks the availability of $ID_{i}$ and checks $C_{4A}\overset{?}{=}h$ ($ID_{i}∥e_{A}∥DID_{A}∥DID_{A}^{new}∥C_{2A}∥SID_{j})$. GWN continues to proceed with the scheme without detection. Unfortunately, the GWN mistakenly believes that he/she is communicating with the legitimate patient $U_{i}$.

Resultingly, the attacker *A* will be successfully confirmed as GWN by user $U_{i}$. Hence, the user impersonation attack is successful.

In the next section, we discuss Wu et al.’s scheme to overcome the weakness of the scheme. Our scheme stores several variables in the database to prevent the vulnerability of Wu et al.

## 5. Proposed Scheme

We propose a new three-factor user authentication scheme for wireless sensor networks in this section. We use three participants: the user $U_{i}$, the gateway node GWN and the sensor node $S_{j}$. The gateway node GWN creates master keys *x*. The user $U_{i}$ and the sensor node $S_{j}$ computes on elliptic curve group $F_{p}$.

We have defined the name of the variable as follows:

- $G_{1},G_{2},G_{3}$: Generator of smart card,
- $MU_{1},MU_{2},MU_{3}$: message sent by user,
- $MG_{1},MG_{2},MG_{3},MG_{4}$: message sent by gateway node,
- $MS_{1},MS_{2},MS_{3}$: message sent by the server node.

Other variables do not have that special meaning.

The proposed scheme is composed as follows: registration phase, login phase, authentication phase, and password/biometrics change phase.

### 5.1. Registration Phase

In this phase, a user $U_{i}$ chooses an identity $ID_{i}$, imprints biometric template $B_{i}$ at the sensor, and then performs the following steps:

#### 5.1.1. User Registration Phase

1. $U_{i}$ selects $ID_{i}$ and $PW_{i}$. imprints $B_{i}$ via a device for biometrics collection and computes Gen ($B_{i}$) = ($R_{i}$, $P_{bi}$) and $HPW_{i}=h$ ($ID_{i}∥PW_{i}∥R_{i}$). Then, he/she sends $ID_{i}$ to GWN secretly.
2. GWN generates a random number $r_{i}$ and computes $GID_{i}=h$ ($ID_{i}∥r_{i}$).
3. GWN computes $G_{i}^{\prime }=h$ ($GID_{i}∥x$), prepares a smart card for $U_{i}$ containing *h* (·), $h_{1}$ (·), *P*, $GID_{i}$ and the fuzzy extractor.
4. GWN stores $ID_{i}$ and $GID_{i}$ in its database and shares it with $U_{i}$. By storing $ID_{i}$ and $GID_{i}$ in the database, Wu et al. [1]’s problems arising from existing $DID_{i}$ can be solved.
5. $U_{i}$ computes $G_{1}=G_{1}^{\prime }\;\;⊕\;\;HPW_{i}$, $G_{2}=h$ ($ID_{i}∥R_{i}∥PW_{i}$) $\;⊕\;\;GID_{i}$ and $G_{3}=h$ ($ID_{i}∥GID_{i}$). $\{G_{1},G_{2},G_{3},h$ (·), $h_{1}$ (·), P} are stored in the smart card.

#### 5.1.2. Sensor Registration Phase

1. GWN selects an identity $SID_{j}$ for each new sensor $S_{j}$, computes $c_{j}=h$ ($SID_{j}∥x$) and sends {$SID_{j}$, $c_{j}$} to $S_{j}$.
2. $S_{j}$ stores *P*, $SID_{j}$ and $c_{j}$ and joins the WSN.

**Figure 1:**
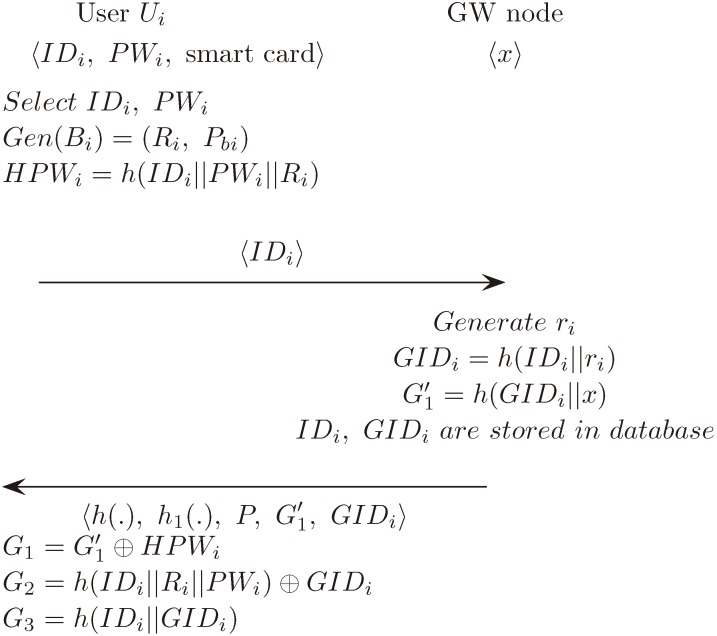
Registration phase of the proposed scheme.

Figure 1 illustrates the registration phase of the proposed scheme.

### 5.2. Login Phase

1. $U_{i}$ inputs $ID_{i}$, $PW_{i}$ and $B_{i}^{\prime }$. The smart card executes Rep ($B_{i}^{\prime }$, $P_{bi}$) = $R_{i}$ and $GID_{i}=G_{2}\;⊕\;h$ ($ID_{i}∥R_{i}∥PW_{i}$). $U_{i}$ checks *h* ($ID_{i}∥GID_{i}$) $\overset{?}{=}G_{3}$. This allows $U_{i}$ to verify whether it has come in correctly.
2. $U_{i}$ generates $e_{i}$ and α. $U_{i}$ computes $HPW_{i}=h$ ($ID_{i}∥PW_{i}∥R_{i}$), $MU_{1}=G_{1}\;⊕\;HPW_{i}\;⊕\;e_{i}$, $MU_{2}=\alpha P$ and $MU_{3}=h$ ($ID_{i}∥e_{i}∥GID_{i}∥MU_{2}∥SID_{j}$).
3. $U_{i}$ sends the message $M_{1}=\{MU_{1}$, $MU_{2}$, $MU_{3}$, $GID_{i}$, $SID_{j}$} to GWN.

**Figure 2:**
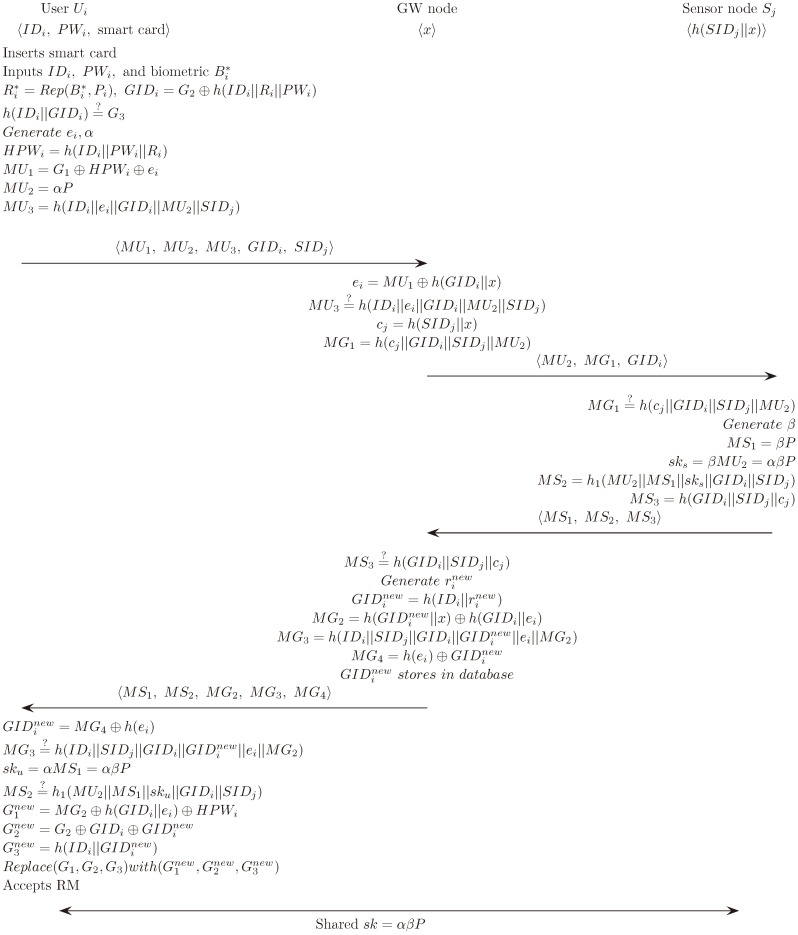
Login and authentication phase of the proposed scheme.

Figure 2 illustrates the login and authentication phase of the proposed scheme.

### 5.3. Authentication Phase

1. GWN finds $ID_{i}$ by using $GID_{i}$ from the database and computes $e_{i}=MU_{1}\;⊕\;h$ ($GID_{i}∥x$). GWN checks the validity of $MU_{3}\overset{?}{=}h$ ($ID_{i}∥e_{i}∥GID_{i}∥MU_{2}∥SID_{j}$). If it fails, the session will be terminated. Otherwise, GWN computes $c_{j}=h$ ($SID_{j}∥x$) and $MG_{1}=h$ ($c_{j}∥GID_{i}∥SID_{j}∥MU_{2}$). When the operation has finished, GWN sends the message $M_{2}=\{MU_{2}$, $MG_{1}$, $GID_{i}\}$ to $S_{j}$.
2. $S_{j}$ checks $MG_{1}\overset{?}{=}h$ ($c_{j}∥GID_{i}∥SID_{j}∥MU_{2}$) with its identity $SID_{j}$. If it is wrong, $S_{j}$ will stop the session. Otherwise, $S_{j}$ selects β∈[1, n−1] and computes $MS_{1}=\beta P$, session key $sk_{s}=\beta MU_{2}$, $MS_{2}=h_{1}$ ($MU_{2}∥MS_{1}∥sk_{s}∥GID_{i}∥SID_{j}$) and $MS_{3}=h$ ($GID_{i}∥SID_{j}∥c_{j}$). It sends message $M_{3}=\{MS_{1}$, $MS_{2}$, $MS_{3}\}$ when all operations have finished.
3. GWN checks $MS_{3}\overset{?}{=}h$ ($GID_{i}∥SID_{j}∥c_{j}$). If it is wrong, the session will be stopped. Otherwise, GWN generates $r_{i}^{new}$ and calculates $GID_{i}^{new}=h$ ($ID_{i}∥r_{i}^{new}$), $MG_{2}=h$ ($GID_{i}^{new}∥x$) ⊕h ($GID_{i}∥e_{i}$), $MG_{3}=h$ ($ID_{i}∥SID_{j}∥GID_{i}∥GID_{i}^{new}∥e_{i}∥MG_{2}$) and $MG_{4}=h$ ($e_{i}$) $⊕\;GID_{i}^{new}$. Finally, GWN sends the message $M_{4}=\{MS_{1}$, $MS_{2}$, $MG_{2}$, $MG_{3}$, $MG_{4}\}$ to $U_{i}$.
4. $U_{i}$ computes $GID_{i}^{new}=MG_{4}\;⊕\;h$ ($e_{i}$) and checks $MG_{3}\overset{?}{=}h$ ($ID_{i}∥SID_{j}∥GID_{i}∥GID_{i}^{new}∥e_{i}∥MG_{2}$). If not, the session will be stopped. $U_{i}$ computes $sk_{u}=\alpha MS_{1}=\alpha \beta P$ and checks $MS_{2}\overset{?}{=}h_{1}$ ($MU_{2}∥MS_{1}∥sk_{u}∥GID_{i}∥SID_{j}$). If it is wrong, $U_{i}$ will stop the session.
5. $U_{i}$ computes $G_{1}^{new}=MG_{2}\;⊕\;h$ ($GID_{i}∥e_{i}$)$⊕HPW_{i}$, $G_{2}^{new}=G_{2}\;⊕GID_{i}\;⊕\;GID_{i}^{new}$ and $G_{3}^{new}=h$$\left(ID_{i}∥GID_{i}^{new}\right)$. Finally, $U_{i}$ substitutes ($G_{1}^{new}$, $G_{2}^{new}$, $G_{3}^{new}$) for ($G_{1}$, $G_{2}$, $G_{3}$) in the smart card, respectively.

### 5.4. Password and Biometrics Change Phase

1. $U_{i}$ inputs $ID_{i}$, $PW_{i}$ and $B_{i}^{\prime }$. The smart card executes Rep ($B_{i}^{\prime }$, $P_{bi}$) = $R_{i}$ and $GID_{i}=G_{2}\;⊕\;h$ ($ID_{i}∥R_{i}∥PW_{i}$). $U_{i}$ checks *h* ($ID_{i}∥GID_{i}$) $\overset{?}{=}G_{3}$. This allows $U_{i}$ to verify whether it has come in correctly.
2. $U_{i}$ is asked to input a new password $PW_{i}^{new}$ and new biometric information $B_{i}^{new}$. The following data are computed: Gen ($B_{i}^{new})=$ ($R_{i}^{new}$, $P_{bi}^{new})$, $HPW_{i}^{new2}=h$ ($ID_{i}∥PW_{i}^{new}∥R_{i}^{new})$, $G_{1}^{new2}=G_{1}\;⊕HPW_{i}\;⊕HPW_{i}^{new2}$, $G_{2}^{new2}=G_{2}\;⊕\;h$ ($ID_{i}∥R_{i}∥PW_{i})\;⊕\;h$ ($ID_{i}∥R_{i}∥PW_{i}^{new2})$. Finally, $U_{i}$ substitutes ($G_{1}^{new2}$, $G_{2}^{new2}$, $P_{bi}^{new}$) for ($G_{1}$, $G_{2}$, $P_{bi}$) in the smart card, respectively.

## 6. Security Analysis of the Proposed Scheme

### 6.1. Formal Security Analysis

The formal security analysis uses an automated analysis tool called ProVerif. ProVerif is an automated tool for analyzing cryptographic protocols that was developed by Bruno Blanchet. Digital signatures, hash functions, signature proofs, etc. are suitable for analyzing an authentication protocol. Recently, many researchers [1,4,24] have verified the authentication in the user authentication protocol using ProVerif. The formal security analysis shows the results of verifying and analyzing the security of the proposed scheme using ProVerif.

We use three channels. We provide the illustration of Table 2. cha is the channel in the registration phase and is used when the user $U_{i}$ and GWN exchange $ID_{i}$ in the registration phase. chc is the channel used by user $U_{i}$ and GWN to exchange messages in the login phase and chb is used when the GWN and Sensor node $S_{j}$ exchange messages in the login phase. Five initial variables were used: $R_{i}$, $ID_{i}$, $ID_{g}$, $SID_{j}$, and $PW_{i}$. $ID_{i}$ and $PW_{i}$ are the personal information made by the user $U_{i}$ when registering. $R_{i}$ is a random string made up of the user’s biometric information. $ID_{g}$ is the identity of the gateway and $SID_{j}$ is the unique string of the sensor node $S_{j}$. *x* is defined as a secret key. *P* is a generator for creating a session key, which is the initial value used in ECC. The concatenate function and the xor function, including the multiplication in ECC and the hash function *h* and $h_{1}$, are defined for the events that indicate the start and end of each.

Table 3 shows the registration phase of the user $U_{i}$ and the process of the login and authentication phase. Table 4 demonstrates the registration phase and the login and authentication phase of the GWN. Table 5 displays the authentication phase of the sensor node $S_{j}$. Table 6 shows the query against the attack with the prover- sive, and Table 7 shows the result for Table 6.

When the code that makes up the scheme is executed, ProVerif prints the following results:

1. RESULT inj-event(EVENT) ==> inj-event(EVENT) is true.
2. RESULT inj-event(EVENT) ==> inj-event(EVENT) is false.
3. RESULT (QUERY) is true.
4. RESULT (QUERY) is false.

The first code means that the event has been verified and the authentication has been successful, while the second code means that the event has not been verified. The third code means that the query was proven and the attack was not successful. When the fourth code is displayed, the query is false, meaning that an attack is possible and the attack induction and tracking is thus displayed.

The ProVerif result of the proposed scheme is shown to be accurate for all events by simulating the result as shown in the figure (see Table 8). Therefore, the proposed scheme is safe from virtual attacker A and the virtual attack has been successfully terminated.

### 6.2. Informal Security Analysis

#### 6.2.1. Privileged Insider Attack

The only value that the user sends in the registration center is the $ID_{i}$. However, their $ID_{i}$ is used after hashing with other values at every subsequent step. It can not be used because it is used as hashed with values that are not exposed to the outside such as $PW_{i}$ or $R_{i}$, $GID_{i}$, $GID_{i}^{new}$, $e_{i}$, $MU_{2}$ and $SID_{j}$, $MG_{2}$, and these values are not exposed. Therefore, it is safe from a privileged insider attack.

#### 6.2.2. Outsider Attack

$U_{i}$’s smart cards include *h* (·), $h_{1}$ (·), *P*, $GID_{i}$, and fuzzy extractors. Information such as session key or $ID_{i}$, which can be a critical value, or information such as a user’s password are all hashed, or can not be extracted because the value can not be extracted from ECC. In addition, IDs and GIDs are kept in the database, and $ID_{i}$ information can not be extracted because $ID_{i}$ are not used directly in the protocol.

#### 6.2.3. Offline ID Guessing Attack

$PW_{i}$ and $ID_{i}$ are not used directly in this phase. They are used through hashing by concatenating them with other variables, so $ID_{i}$ and $PW_{i}$ can not be directly obtained from public information. Therefore, $ID_{i}$ and $PW_{i}$ can not be obtained using login request messages $MU_{1}$, $MU_{2}$, $MU_{3}$, $GID_{i}$, and $SID_{j}$. Since $ID_{i}$ and $GID_{i}$ are combined and stored in the database, it is impossible to extract the $ID_{i}$ from the protocol.

#### 6.2.4. Online ID Guessing Attack

$ID_{i}$ and $PW_{i}$ are not directly used in the phase so the attacker can not guess the $ID_{i}$s or passwords of others. It is impossible to retrieve a user’s $ID_{i}$ in the protocol because the IDs and GIDs are stored in the database, and $ID_{i}$ is found by searching the database.

#### 6.2.5. Session Key Disclosure Attack

The session key should be computed as β or α when knowing αP or βP with αβP. Neither β nor α are known to the user or the sensor node, so it is impossible to know the session key unless it is a user or a sensor node.

#### 6.2.6. User Impersonation Attack

After the $ID_{i}$ is found in the database using the GID, $e_{i}=MU_{1}+h$ ($GID_{i}\left||x\right)$ is calculated in order to compare the $MU_{3}$ and *h* ( $ID_{i}∥e_{i}∥GID_{i}∥MU_{2}∥SIDj_{)}$. One can never be accepted as a specific user without knowing the ID and GID pair. Therefore, a User Impersonation Attack is impossible.

#### 6.2.7. Server Impersonation Attack

The server is identified in $MS_{3}$ = *h* ($GID_{i}∥SID_{j}∥c_{j})$. $c_{j}=h$ ($SID_{j}∥x)$ and *x* is the secret key. Therefore, it is necessary to know the $c_{j}$ calculated by the secret key other than the $GID_{i}$ and the $SID_{j}$ included in the message in order to authenticate the server and $c_{j}$ is not used alone and $MG_{1}$ = *h* ($c_{j}∥GID_{i}∥SID_{j}∥MU_{2})$, $MS_{3}=h$ ($GID_{i}∥SID_{j}∥c_{j})$ and other values. In addition, the value *x* in the destination $c_{j}=h$ ($SID_{j}∥x)$ can not be determined because it is always used by hashing with $SID_{j}$.

#### 6.2.8. User Anonymity

In the login process, the user gives $MU_{1}$, $MU_{2}$, $MU_{3}$, $GID_{i}$, and $SID_{j}$ to the GWN. In this case, $GID_{i}$ = $G_{2}+h$ ($ID_{i}∥R_{i}∥PW_{i}$) is continuously changed by the random number $R_{i}$. Since $ID_{i}$ is used by hashing, one cannot guess $ID_{i}$ through $MU_{1}$, $MU_{2}$, $MU_{3}$, $GID_{i}$, and $SID_{j}$.

#### 6.2.9. Forward Secrecy and Backward Secrecy

Because of the nature of ECCDH, we can not find αP and βP through αβP, we can not find αβP through αP and βP, and we can not find α through *P* and αP.

## 7. Performance Analysis of the Proposed Scheme

Four symbols in total are used to analyze performance. $T_{m}$ is the time of the multiplicative operation used in ECC. This takes the most time in our scheme. $T_{Rep}$ assumes that it is equal to $T_{m}$, the time to check for a match when recognizing the user’s biometric $B_{i}^{*}$. $T_{s}$ means time in symmetric encryption or decryption. Finally, $T_{h}$ means the time it takes to use the hash function. These are listed in Table 9.

The authors [26] measured the approximate execution time of each cryptographic operation under the following conditions:

- CPU: Intel(R) Core(TM)2T6570 2.1 GHz,
- Memory: 4 G,
- OS: Win7 32-bit,
- Software: Visual C++ 2008,
- MIRACL C/C++ Library,
- Security level: 160-bit point in $F_{p}$,
- 1024-bit in a cyclic group, AES and SHA-1.

The proposed scheme produced the best results in time among all the three factor user authentication schemes using ECC (see Table 10).

## 8. Conclusions

Many user authentication schemes have been proposed for wireless sensor networks, but they have serious security flaws, respectively. Recently, Wu et al. also proposed a three-factor user authentication scheme, which is looking promising. However, we discovered vulnerabilities in the configuration of their scheme and proposed a new scheme to address the discovered issues. Finally, we provide security and performance analysis between the Wu et al. scheme and our proposed protocol, and provide formal analysis based on the ProVerif. The security and performance of the proposed scheme are significantly better than the existing user authentication schemes. Our scheme is not very fast yet. In the future, we will study the WSN protocol, which is safer, simpler and faster.

## Figures and Tables

**Table 1:** Notations used in this paper.

Notations	Description
$U_{i}$	The *i*-th user
$S_{j}$, $SID_{j}$	A *j*-th sensor and its identity
$ID_{i}$	$U_{i}$’s identification
$PW_{i}$	Password of $U_{i}$
$B_{i}$	$U_{i}$’s Biometric information summarized
A	An evil-minded attacker
*x*	Secret key of GWN
$r_{i}$	Random number generated by $U_{i}$
*h* (·), $h_{1}$ (·)	One-way hash function
X∥Y	Concatenation operator
⊕	Bitwise XOR operator
*E* ($F_{p})$	A group of points on a finite field $F_{p}$ elliptic curve
*P*	A point generator in $F_{p}$ with a large prime order *n*
*G*	A cyclic addition group under *P* as a generator
$sk_{u}$, $sk_{s}$	The session key generated by $U_{i}$ and $S_{j}$, respectively.

**Table 2:** Define values and functions.

(*—-channels—-*)
free cha:channel [private].
free chb:channel.
free chc:channel.
(*—-constants—-*)
free Ri:bitstring [private].
free IDi:bitstring [private].
free IDg:bitstring.
free SIDj:bitstring.
free PWi:bitstring [private].
(*—-secret key—-*)
free x:bitstring [private].
(*—-shared key—-*)
free P:bitstring [private].
(*—-functions—-*)
fun concat(bitstring, bitstring):bitstring.
fun xor(bitstring, bitstring):bitstring.
fun h(bitstring):bitstring.
fun h1(bitstring):bitstring.
fun mult(bitstring, bitstring):bitstring.
equation forall a:bitstring, b:bitstring; mult(a, b) = mult(b, a).
equation forall a:bitstring, b:bitstring; xor(xor(a, b), b) = a.
(*—-events—-*)
event beginUi(bitstring).
event endUi(bitstring).
event beginGWN(bitstring).
event endGWN(bitstring).
event beginSj(bitstring).
event endSj(bitstring).

**Table 3:** $U_{i}$ protocol.

(*—-Ui process—-*)
let Ui =
let HPWi = h(concat(concat(IDi, PWi), Ri)) in
out(cha,(IDi));
in(cha,(XGIDi:bitstring));
let G1’ = h(concat(XGIDi, x)) in
let G1 = xor(G1’, HPWi) in
let G2 = xor(h(concat(concat(IDi, Ri), PWi)), XGIDi) in
let G3 = h(concat(IDi, XGIDi)) in
event beginUi(IDi);
new ei:bitstring;
new alpha:bitstring;
let GIDi = xor(G2, h(concat(concat(IDi, Ri), PWi))) in
if h(concat(IDi, XGIDi)) = G3 then
let HPWi = h(concat(concat(IDi, PWi), Ri)) in
let MU1 = xor(xor(G1, HPWi), ei) in
let MU2 = mult(alpha, P) in
let MU3 = h(concat(concat(IDi, ei), concat(concat(XGIDi, MU2), SIDj))) in
out(chc,(MU1, MU2, MU3, GIDi, SIDj));
in(chc,(XXMS1:bitstring, XXMS2:bitstring,
XMG2:bitstring, XMG3:bitstring, XMG4:bitstring));
let GIDinew = xor(XMG4, h(ei)) in
if XMG3 = h(concat(concat(IDi, SIDj),
concat(concat(GIDi, GIDinew), concat(ei, XMG2)))) then
let sku = mult(alpha, XXMS1) in
if XXMS2 = h1(concat(concat(MU2, XXMS1),
concat(concat(sku, GIDi), SIDj))) then
let G1new = xor(XMG2, xor(h(concat(GIDi, ei)), HPWi)) in
let G2new = xor(G2, xor(GIDi, GIDinew)) in
let G1 = G1new in
let G2 = G2new in
event endUi(IDi).

**Table 4:** GWN protocol.

(*—-GWN process—-*)
let GWN =
in(cha, (XIDi:bitstring));
new ri:bitstring;
let GIDi = h(concat(XIDi, ri)) in
let G1’ = h(concat(GIDi, x)) in
out(cha, (GIDi));
in(chc, (XMU1:bitstring, XMU2:bitstring, XMU3:bitstring, XGIDi:bitstring, XSIDj:bitstring));
event beginGWN(IDg);
let ei = xor(XMU1,h(concat(XGIDi, x))) in
if XMU3 = h(concat(concat(XIDi, ei),
concat(concat(XGIDi, XMU2), XSIDj))) then
let cj = h(concat(XSIDj, x)) in
let MG1 = h(concat(concat(cj, XGIDi), concat(XSIDj, XMU2))) in
out(chb, (XMU2, MG1, XGIDi));
in(chb, (XMS1:bitstring, XMS2:bitstring,
XMS3:bitstring));
if XMS3 = h(concat(concat(XGIDi, XSIDj), cj)) then
new rinew:bitstring;
let GIDinew = h(concat(XIDi, rinew)) in
let MG2 = xor(h(concat(GIDinew, x)), h(concat(XGIDi, ei))) in
let MG3 = h(concat(concat(XIDi, XSIDj), concat(concat(XGIDi, GIDinew), concat(ei, MG2)))) in
let MG4 = xor(h(ei), GIDinew) in
out(chc, (XMS1, XMS2, MG2, MG3, MG4));
event endGWN(IDg).

**Table 5:** $S_{j}$ protocol.

(*—-Sj process—-*)
let Sj =
in(chb, (XXMU2:bitstring, XMG1:bitstring, XXGIDi:bitstring));
event beginSj(SIDj);
let scj = h(concat(SIDj, x)) in
if XMG1 = h(concat(concat(scj, XXGIDi), concat(SIDj, XXMU2))) then
new beta:bitstring;
let MS1 = mult(beta, P) in
let sks = mult(beta, XXMU2) in
let MS2 = h1(concat(concat(XXMU2, MS1), concat(concat(sks, XXGIDi), SIDj))) in
let MS3 = h(concat(concat(XXGIDi, SIDj), scj)) in
out(chb, (MS1, MS2, MS3));
event endSj(SIDj).

**Table 6:** Queries.

(*—-queries—-*)
query attacker(P).
query id:bitstring; inj-event(endUi(id)) ==> inj-event(beginUi(id)).
query id:bitstring; inj-event(endGWN(id)) ==> inj-event(beginGWN(id)).
query id:bitstring; inj-event(endSj(id)) ==> inj-event(beginSj(id)).
process
((!Ui)|(!GWN)|(!Sj))

**Table 7:** Output of queries.

RESULT inj-event(endSj(id)) ==> inj-event(beginSj(id) is true.
RESULT inj-event(endGWN(id_12209)) ==> inj-event(beginGWN(id_12209) is true.
RESULT inj-event(endUi(id_25655)) ==> inj-event(beginUi(id_25655) is true.
RESULT not attacker(P[]) is true.

**Table 8:** Performance comparison.

Features	Wu et al. [1]	Park et al. [3]	Park et al. [25]	Ours
Defence of privileged insider attack	O	O	O	O
Defence of outsider attack	X	X	X	O
Defence of offline ID guessing attack	O	O	O	O
Defence of online ID guessing attack	X	X	X	O
Defence of session key disclosure attack	O	O	O	O
Defence of user impersonation attack	X	X	O	O
Defence of server impersonation attack	O	X	O	O
User anonymity	X	O	X	O
Forward secrecy and backward secrecy	O	O	O	O

**Table 9:** Notations of time symbol.

Symbol	Meaning	Time (ms)
$T_{m}$	time of multiplication in Field	7.3529 [26]
$T_{Rep}$	time of Rep	=$T_{m}$ [16]
$T_{s}$	time of symmetric encryption or decryption	0.1303 [26]
$T_{h}$	time of hash operation	0.0004 [26]

**Table 10:** Performance comparison.

	Wu et al. [1]	Park et al. [3]	Park et al. [25]	Ours
User $U_{i}$	10$T_{h}$ + 1$T_{Rep}$ + 2$T_{m}$	6$T_{h}$ + 1$T_{Rep}$ + 2$T_{m}$	10$T_{h}$ + 1$T_{Rep}$ + 2$T_{m}$	8$T_{h}$ + 1$T_{Rep}$ + 2$T_{m}$
(ms)	= 22.0627	= 22.0611	= 22.0627	= 22.0619
GWN	10$T_{h}$	7$T_{h}$ + 2$T_{e}$	11$T_{h}$	10$T_{h}$
(ms)	= 0.004	= 0.2634	= 0.0044	= 0.004
Sensor node $S_{j}$	2$T_{h}$ + 2$T_{m}$	6$T_{h}$ + 2$T_{m}$ + 1$T_{e}$	4$T_{h}$ + 2$T_{m}$	3$T_{h}$ + 2$T_{m}$
(ms)	= 14.7066	= 14.8385	= 14.7074	= 14.707
Total costs	22$T_{h}$ + 4$T_{m}$ + 1$T_{Rep}$	19$T_{h}$ + 4$T_{m}$ + 3$T_{e}$ + 1$T_{Rep}$	25$T_{h}$ + 4$T_{m}$ + 1$T_{Rep}$	21$T_{h}$ + 4$T_{m}$ + 1$T_{Rep}$
(ms)	= 36.7733	= 37.163	= 36.7745	= 36.7729

## Appendix A

**Table A1:** Explanation of each abbreviation.

Notations	Description
WSN	Wireless sensor network
RSA	A public-key encryption technology developed by Ron Rivest, Adi Shamir, and Leonard Adleman
ECC	Elliptic curve cryptosystem created by Victor S. Miller and Neal Koblitz
Gen	A probabilistic generation function for which the biometrics *B* returns a string α and a string β
Rep	A function that restore β to α and any vector $B^{*}$ close to *B*
*B*	A vector with biometric information
$B^{*}$	Any vector $B^{*}$ close to *B*
GWN	Gateway node
ProVerif	An analysis tool for protocol verification
